# Composition and distribution characteristics of acetic acid-related bacteria during the fermentation process of strong-flavor baijiu

**DOI:** 10.3389/fmicb.2025.1603412

**Published:** 2025-07-17

**Authors:** Mandlaa Mandlaa, Mengyu Liu, Guojun Ren, Hui Qu, Hui Wang, Fei Ma, Min Zhang, Jing Guo, Zhongjun Chen, Ziyu Sun

**Affiliations:** ^1^College of Food Science and Engineering, Inner Mongolia Agricultural University, Hohhot, China; ^2^Inner Mongolia Hetao Liquor Group Co., Ltd., Bayannur, China

**Keywords:** strong-flavor baijiu, ethyl acetate, acetic acid, composition, source tracking, bacteria

## Abstract

Ethyl acetate (EA) has an important impact on the quality of Strong-flavor Baijiu (SFB) and is synthesized from acetic acid (AA) and ethanol by microorganisms during the fermentation process. However, the composition and distribution characteristics of AA-related microorganisms are unclear in the SFB production process. In this study the changes of bacterial community and concentration of AA during the fermentation process (7-67d) and different season fermentation (spring and summer) was investigated, and the composition of AA-related bacteria was analyzed to reveal the distributional characteristics of AA-related bacteria in brewing environment (Daqu, raw materials, pit mud, ground and tools). The results showed that the concentration of AA increased during the fermentation process, and AA in summer was significantly higher than in spring (*p* < 0.05). A total of 23 genera were significantly related to AA (*p* < 0.05), and most of them were also significantly related (*p* < 0.05) to the dominant genus, reducing sugar, moisture, and acidity. Moreover, *Pantoea* (negatively related to AA) mainly comes from Daqu^F^ and was the dominant genus during the fermentation process. *Saccharopolyspora, Lactococcus*, and *Streptomycetaceae* had low abundance and a negative relationship with bacteria mainly found in Daqu^L^ and pit mud. *Unclassified_Bacilli* had a positive relationship with AA and mainly came from raw material. Ground and tools can provide seven genera with low relative abundance. This study lays a foundation for establishing methods to regulate AA in SFB.

## 1 Introduction

Baijiu, a traditional distilled alcoholic beverage of China (Wang L. et al., 2024), can be categorized into 12 types (strong-flavor, sauce-flavor, light-flavor, and others) based on the flavor characteristic (Zhang et al., 2022). Among the 12 types, strong-flavor Baijiu (SFB) dominates the market and shares half or more amount of the Baijiu consumption in China (Wang Y. et al., 2024). It is reported that the aroma characteristic of SFB, the strong flavor of muddy pit, and soft-sweet taste (Zou et al., 2018), is mainly formed by ethyl caproate combined with ethyl acetate (EA), ethyl butyrate, ethyl lactate, and other aroma components (Li et al., 2020; Liu et al., 2017; Xu S. et al., 2022). Other than the content of ethyl caproate itself, the proportion of ethyl caproate and EA is seen as a key factor in keeping the flavor characteristic of SFB (Qian et al., 2021) and the optimum proportion should be stabilized at 1:(0.5–0.6) (Wei et al., 2020). If the content of EA is excessive and the proportion of ethyl caproate is disproportionate, the flavor characteristic of SFB would be weakened, and the productivity of SFB would be decreased (Sun et al., 2016). The synergistic effect of an appropriate amount of EA with other esters, such as ethyl hexanoate and ethyl lactate, can provide the complex aroma of SFB. In the national standard for SFB of China (GB/T 10781.1–2021), the total ester content and ethyl hexanoate content are used as important indicators for evaluating SFB quality, while no requirements are specified for the EA content. This indicates that an excessive amount of EA has no positive effect on the quality of SFB. Therefore, it is important to control the formation of EA during the fermentation process of SFB.

Based on the literature, EA can be synthesized via direct esterification and an enzymatic esterification reaction during the fermentation process of Baijiu (Song et al., 2019). Compared to the enzymatic esterification reaction, the synthesis of EA by direct esterification reaction from acetic acid and ethanol requires a long reaction time (Kruis et al., 2019; Li et al., 2017). The enzymatic esterification reaction by esterases secreted by microorganisms in extracellular (Li et al., 2022) is considered the primary pathway for synthesizing EA in the Baijiu fermentation process (Xu Y. et al., 2022). During the fermentation process of Baijiu, the accumulation of acetic acid (AA) and ethanol is the basis for synthesizing EA, and it is found that the EA synthesis is influenced by the concentration of substrate (acetic acid and ethanol). When the concentration of AA is 25 g/L, the activity of esterases is the highest (Xing et al., 2018). Consequently, the content of AA is a key factor in impacting the synthesis of EA, and the study of AA synthetic pattern is important to regulate the EA during the fermentation process of Baijiu.

The synthesis of AA during the fermentation process of Baijiu is significantly influenced by the conditions of fermentation and the microorganism involved during the fermentation process (De Filippis et al., 2018). It has been demonstrated that the oxygen content, aging of the pit, and the shorter storage time of Daqu in SFB fermentation can promote the growth of *Acetobacter* and produce excessive AA (Zheng and Han, 2016). However, feeding at a lower temperature can inhibit the reproduction of *Acetobacter* and *Lactobacillus*, and reduce the production of AA in Baijiu fermentation (Lai et al., 2021). Moreover, inoculation of some microorganisms (*Clostridium tyrobutyricum*) related to AA synthesis can regulate AA synthesis in SFB fermentation (Zhou et al., 2022).

The open production process leads to multiple sources, Raw Material (RM), Daqu (DQ), Pit Mud (PM), Ground (GE), and Tools (TL), of microorganisms involved in the fermentation process of Baijiu (Zhang et al., 2005) and a variety of microorganisms related to AA synthesis (Jiao et al., 2023; Xie et al., 2022).

Therefore, clarifying the composition of AA-related microorganisms and the characteristics of their distribution in the brewing environment is of great significance for establishing acetic acid regulation methods for SFB production. In this study, headspace solid-phase microextraction coupled with gas chromatography–mass spectrometry (HS-SPME-GC–MS) was applied to investigate the concentration of AA and EA. High-throughput sequencing technology was applied to investigate the diversity and composition of bacteria in different samples from the fermentation process (7–67 days), different season fermentation (spring and summer) and in samples of RM, DQ, PM, GE and TL of SFB. In addition, the changing pattern of AA and the diversity of bacteria under the two sampling methods were combined to explore the composition and distribution of AA-related bacteria during the fermentation process of SFB.

## 2 Materials and methods

### 2.1 Collection of samples

All samples involved in this study were collected from Inner Mongolia Hetao Liquor Group Co., Ltd. During the fermentation process, collections were conducted on days 7, 14, 21, 28, 42, 55, and 67 of the fermentation process. Triplicate cellar pits were sampled at each time point, with stratified samples (upper, middle, and lower layers) retrieved from each pit and then pooled to form one sample. In different seasons, fermented grains were collected at the end of fermentation in March and July. Ten cellar pits were sampled per season, and stratified samples from each pit were pooled into one sample. Raw materials, Daqu, and pit mud were sampled thrice, respectively. Tool and floor surfaces were swabbed with 0.01% PBS buffer (three replicates), and swabs were transferred to sterile centrifuge tubes. All samples were immediately cryopreserved at −80°C after collection.

### 2.2 Analytical methods

#### 2.2.1 Determination of AA and EA of fermented grains

HS-SPME-GC–MS was applied to determine the content of AA and EA in the fermented grains (FG). Firstly, 2 g FG and 20 μL of 5 g/L 2-octanol (internal standard) were added into a headspace vial and extracted by 80 μm/10 mm DVB/CAR/PDMS at 50°C for 45 min. After extraction, the extract was determined by GC–MS, and the parameters of GC–MS are as follows: the temperature of the column (DB-WAX, 60 m × 0.25 mm × 0.25 μm) was maintained at 50°C for 3 min and then increased to 230°C at a rate of 6°C/min. The temperature of the inlet and detector was 250°C, and the mass spectrometry conditions were the same as those found in the study by Qiao et al. (2023).

#### 2.2.2 Determination of moisture, acidity, and reducing sugar of FG

Moisture: FG (5 g) was dried at 105°C until its mass became constant. Calculate the water content based on the mass before and after drying (Ge et al., 2024). Acidity: The acidity was determined by the acid–base titration method (Xia et al., 2024). Reducing sugar: 0.8 mL of distilled water and 1 mL of DNS were added into 0.2 mL solution of the sample and diluted the mixed solution to 10 mL. The absorbance (520 nm) of the final solution was determined, and the reducing sugar content was calculated based on the standard curve (Lv, 2023).

#### 2.2.3 Extraction of genome DNA and high-throughput sequencing of 16S rRNA

E. Z. N. A™ Mag-Bind Soil DNA Kit was used to extract the genomic DNA of FG according to the instruction manual. The V3-V4 region of bacterial 16S rRNA was amplified by primers 341F and 805R, and the amplicon was sequenced by Illumina MiSeq Shanghai Sangong Bioengineering Company Limited. After sequencing, the raw data were processed according to QIIME 2 (Caporaso et al., 2010) and annotated after delineation of OUT (>97% similarity; Edgar, 2013). Chao1 and Shannon index were calculated via Mothur^1^ to indicate the bacterial richness and diversity of the samples (Dai et al., 2020).

#### 2.2.4 Bioinformatics, statistical analysis, and plotting

Principal component analysis (PCA) was displayed to evaluate the ecological distances of different samples based on weighted UniFrac distances by using the R vegan package (Wang et al., 2017). Line Discriminant Analysis Effect Size (LEfSe) was used to determine the differentially abundant taxa between different groups (Segata et al., 2011). One-way analysis of variance (ANOVA) was applied to evaluate the differences between the two groups, and *p* < 0.05 was considered to indicate a significant difference between the two groups. Pearson correlation analysis was applied to evaluate the correlation, and it was considered a significant correlation if |R| > 0.5 and *p* < 0.05. FEAST of traceability analysis^2^ was applied to analyze the source of the FG bacteria (Shenhav, 2019). Origin 2022, R 4.3.0, and Gephi 10.1 were applied to plot the figure.

## 3 Results

### 3.1 Changes of AA, EA, moisture, acidity, and reducing sugar in FG during fermentation process and different seasons

**Figure 1:**
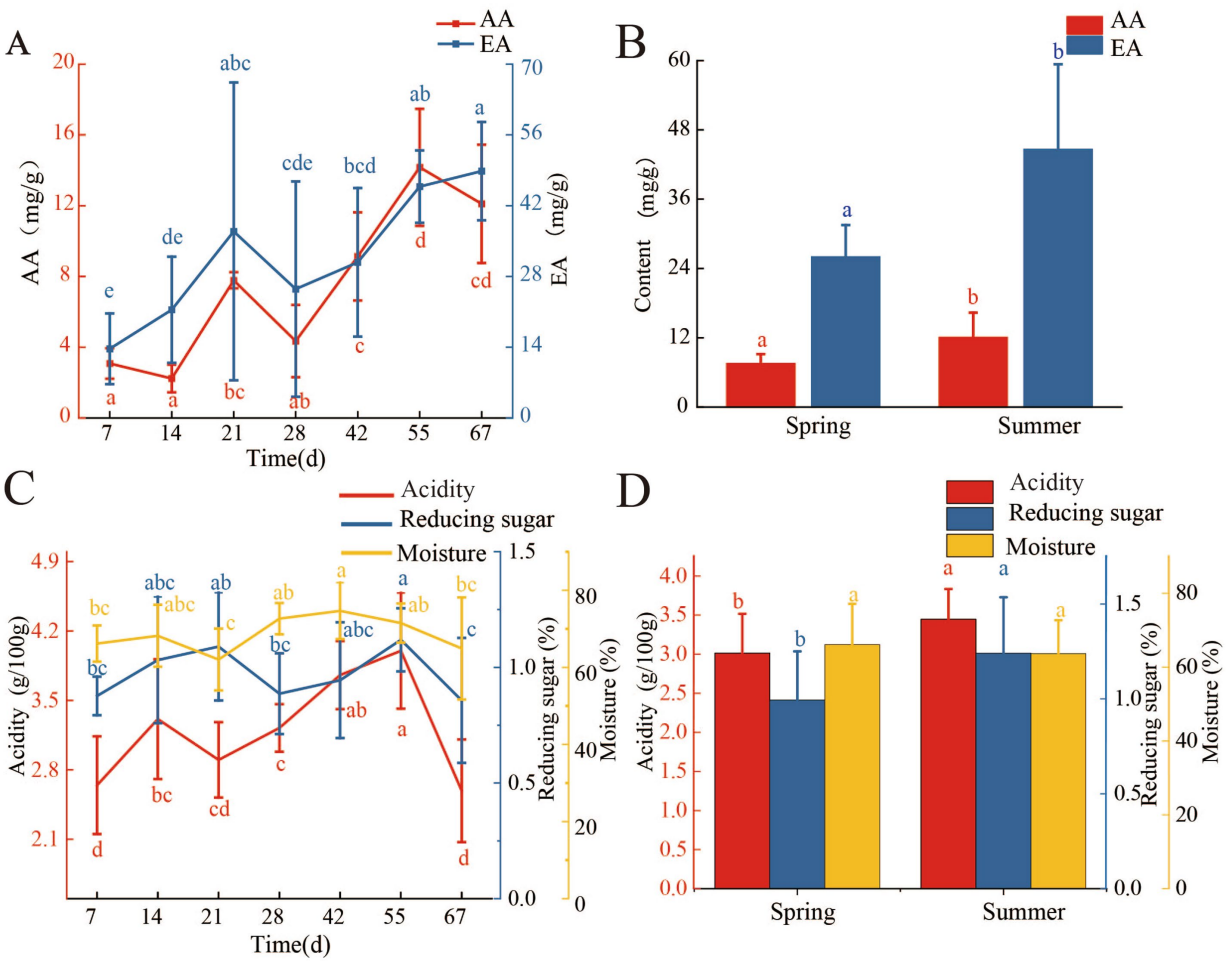
Changes of AA, EA, moisture, acidity, and reducing sugar during fermentation process and in different seasons. **(A)** Changes of AA and EA during fermentation process. **(B)** Changes of AA and EA in different seasons. **(C)** Changes in moisture, acidity, and reducing sugar during the fermentation process. **(D)** Changes in moisture, acidity, and reducing sugar in different seasons.

During the fermentation process, the concentration of AA in FG gradually increased (Figure 1A). The highest concentration of AA (14.17 ± 3.31 mg/g) was found on the 55th day of fermentation, and it was significantly higher than that on the 7th day of fermentation (3.08 ± 0.86 mg/g, *p* < 0.05). In the later period of fermentation (55–67 days), the concentration of AA had no significant changes (*p* < 0.05). The changing pattern of EA in FG was the same as AA, and the concentration of EA increased gradually during the fermentation process (Figure 1A). After 67 days of fermentation, the concentration of EA in FG was 48.83 ± 9.73 mg/g, and it was 2.56 times higher than that on the 7th day of FG. The acidity, moisture, and reducing sugar concentration in FG were changed during the fermentation process (Figure 1C). The Acidity of FG significantly increased from 2.6 g/100 g (7th day) to 4 g/100 g (55th day, *p* < 0.05) and then dropped to 2.6 g/100 g at 67 days of fermentation. Compared with the changes of acidity, the variation of moisture and reducing sugar concentration in FG was smoother, as shown in Figure 1C. The significantly higher moisture (74.66%) and reducing sugar concentration (1.12%) were found at 42nd and 55th days of FG, respectively (*p* < 0.05).

Fermentation in different seasons also had a significant impact on the concentration of AA, EA, reducing sugar, and acidity of FG (Figures 1B,D). The concentration of AA was significantly higher in summer FG (12.07 ± 4.27 mg/g) than spring (7.52 ± 1.64 mg/g, 67th day) (*p* < 0.05; Figure 1B), and temperature may be a crucial factor affecting the accumulation of AA during the fermentation process. The summer FG had a higher concentration of EA (44.62 ± 14.76 mg/g), acidity (3.44 g/100 g), and reducing sugar (1.24 g/100 g) than spring, respectively (Figure 1D; *p* < 0.05). However, moisture content showed no significant difference between spring and summer FG (*p* > 0.05).

### 3.2 Diversity and composition of bacteria in FG (fermentation process and different seasons), RM, DQ^L^, DQ^F^, PM, GE, and TL

#### 3.2.1 *α*-Diversity of bacteria in FG (fermentation process and different seasons), RM, DQ^L^, DQ^F^, PM, GE, and TL

**Figure 2:**
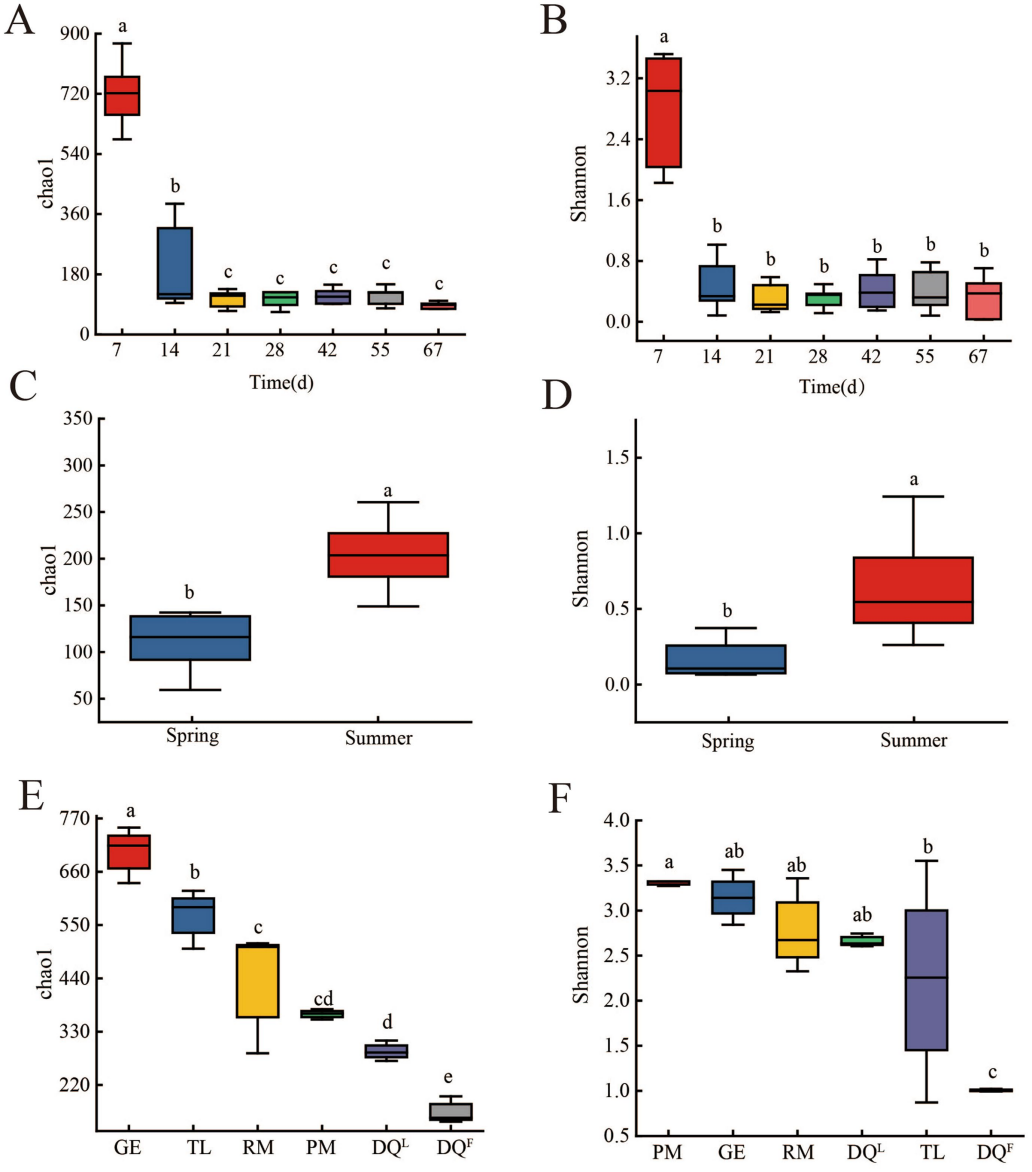
*α*-Diversity of bacteria in FG (fermentation process and different seasons), Raw Material (RM), Daqu (DQ^L^, DQ^F^), Pit Mud (PM), Ground (GE) and Tools (TL). **(A)** Chao1 index of bacteria in FG of the fermentation process. **(B)** Shannon index of bacteria in FG of the fermentation process. **(C)** Chao1 index of bacteria in FG of different seasons. **(D)** Shannon index of bacteria in FG of different seasons. **(E)** Chao1 index of bacteria in RM, DQ^L^, DQ^F^, PM, GE and TL. **(F)** Shannon index of bacteria in RM, DQ^L^, DQ^F^, PM, GE, and TL.

Chao1 and Shannon index were applied to characterize the richness and diversity of bacterial community in FG (fermentation process and different seasons), RM, DQL, DQF, PM, GE, and TL and the results were shown in Figure 2. During the fermentation process, Chao1 and Shannon index of 7th day’s FG was significantly higher than others (Figures 2A,B; *p* < 0.05). It indicated that bacterial richness and diversity were higher in the early period of fermentation process and declined after the 14th day of fermentation. Moreover, fermentation in different seasons also had a significant effect on the Chao1 and Shannon indices of FG. Chao1 and Shannon indices of FG in summer were significantly higher than in spring (Figures 2C,D; *p* < 0.05). It indicated that the richness and diversity of bacteria in summer FG were higher than in spring.

Moreover, the bacterial diversity of PM, GE, DQ^L^, DQ^F^, TL, and RM was analyzed. The results are shown in Figures 2E,F. The Chao1 index decreased in the order of GE, TL, RM, PM, DQ^L^6, DQ^F^, and GE (700.59), with the highest Chao1 index, and DQ^F^ (164.53) having the lowest Chao1 index (*p* < 0.05). It indicated that the richness of bacteria was gradually decreased in the order of GE, TL, RM, PM, DQ^L^, DQ^F^, with GE having the highest richness of bacteria, whereas DQ^F^ had the lowest richness of bacteria. However, the Shannon index of the six sources showed a different pattern from the Chao1 index (Figure 2F). The Shannon index of RM, GE, PM, and DQ^L^ was significantly higher than that of TL and DQ^F^ (*p* < 0.05). It indicated that the richness of bacteria in M, GE, PM, and DQ^L^ was higher than that of TL and DQ^F^.

#### 3.2.2 Bacterial composition of FG (fermentation process and different seasons), RM, DQ^L^, DQ^F^, PM, GE, and TL

**Figure 3:**
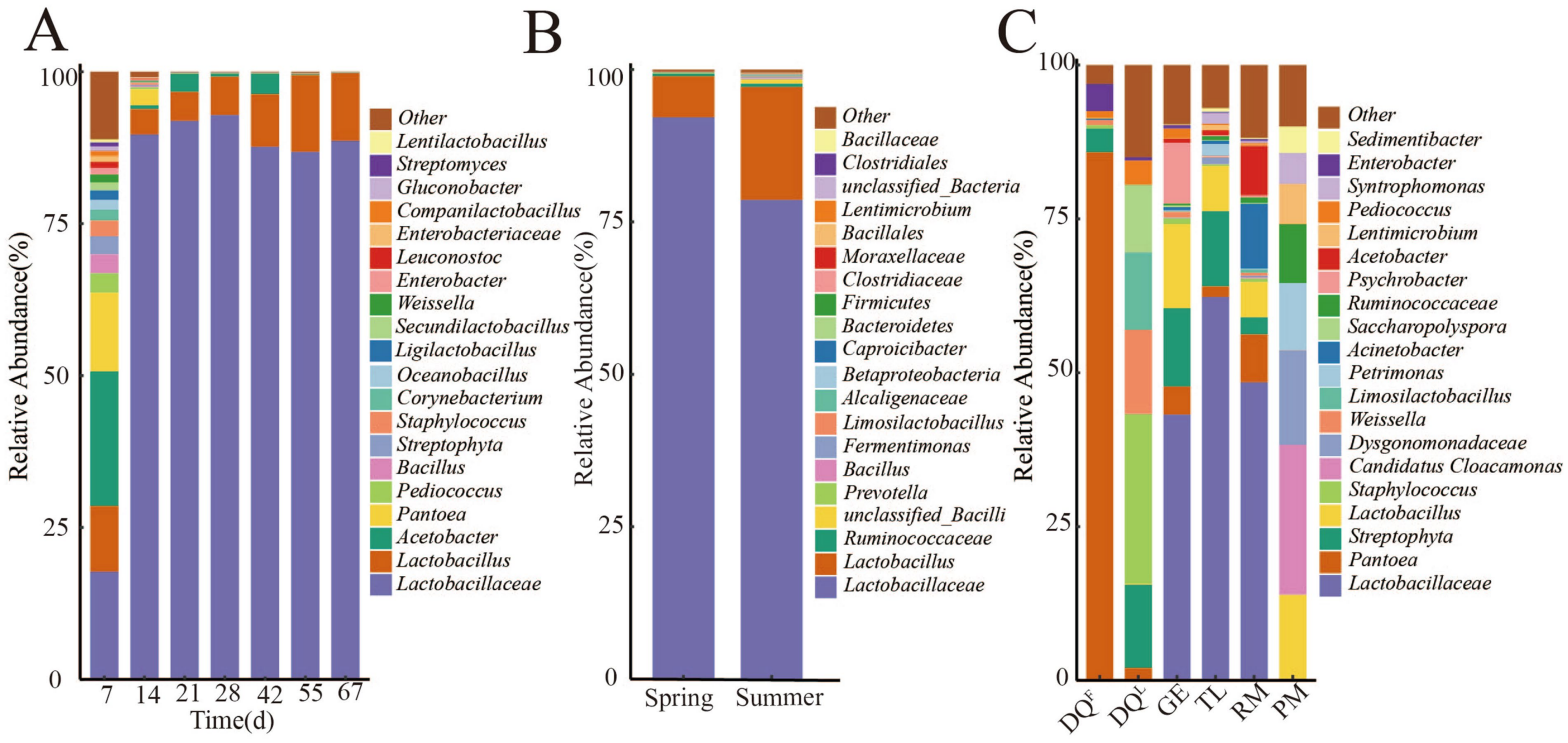
Composition of bacteria in FG (fermentation process and different seasons), Raw Material (RM), Daqu (DQ^L^, DQ^F^), Pit Mud (PM), Ground (GE) and Tools (TL). **(A)** Composition of bacteria in FG of fermentation process. **(B)** Composition of bacteria in FG of different seasons. **(C)** Composition of bacteria in RM, DQ^L^, DQ^F^, PM, GE and TL.

To understand the bacterial composition of FG (fermentation process and different seasons), RM, DQ^L^, DQ^F^, PM, GE, and TL, the relative abundance of genus level of them was counted and shown in Figure 3. The results showed that *Acetobacter* (22.1%), *Lactobacillaceae* (17.7%), *Pantoea* (12.9%), *Lactobacillus* (10.8%), *Pediococcus* (3.2%), *Bacillus* (3.1%) and *Weissella* (1.3%) were the dominant genus (relative abundance >1%) of the early stage of fermentation (7th day) and the genus of *Lactobacillaceae* and *Lactobacillu*s became the dominate after 14 days fermentation. At the end of fermentation (67th day), the relative abundance of *Lactobacillaceae* and *Lactobacillu*s reached 88.6 and 11.14%, respectively. The relative abundance of *Acetobacter* decreased from 14 days and fluctuated in the 21–42 days of fermentation, then decreased to < 1% at the end of fermentation. In addition, the relative abundance of *Pantoea*, *Pediococcus*, *Bacillus*, and *Weissella* also decreased from the middle of the fermentation process and reached < 1% at the end of fermentation (Figure 3A).

In different seasons, *Lactobacillaceae* and *Lactobacillus* were the absolute dominant genus, but their abundance varied in different seasons as shown in Figure 3B. The relative abundance of *Lactobacillaceae* was higher in spring (92.2%) than in summer (78.6%), while the relative abundance of *Lactobacillus* was higher in summer (18.9%) than in spring (6.8%). In addition, the relative abundance of *Prevotella*, *Ruminococcaceae*, and *Bacillus* also varied in different seasons.

Furthermore, the bacterial composition in RM, DQ^L^, DQ^F^, PM, GE, and TL was analyzed, and the results are shown in Figure 3C. The composition of bacteria exhibited considerable variation in RM, DQ^L^, DQ^F^, GE, PM, and TL. The dominant genera in PM were *Candidatus Cloacamonas* (24.3%), *Dysgonomonadaceae* (15.3%), *Petrimonas* (10.9%), *Ruminococcaceae* (9.6%), *Lentimicrobium* (6.5%), *Syntrophomonas* (5.0%), *Sedimentibacter* (4.3%), and *Aminobacterium* (3.2%). The absolutely dominant genus of DQ^F^ was *Pantoea* (85.8%), *Staphylococcus* (27.6%), *Weissella* (13.7%), *Limosilactobacillus* (12.5%), and *Saccharopolyspora* (11.1%), which were the dominant genera of DQ^L^. The dominant genera in GE were *Lactobacillaceae* (43.17%), *Lactobacillus* (13.67%), and *Streptophyta* (12.74%), while the absolute dominant genera in TL were *Lactobacillacea*e (62.30%) and *Streptophyta* (12.21%). *Lactobacillaceae* (48.43%), *Acinetobacter* (10.53%), and *Acetobacter* (8.01%) were the dominant genera in RM.

#### 3.2.3 *Β*-Diversity and differential genus in FG of the fermentation process and different seasons

**Figure 4:**
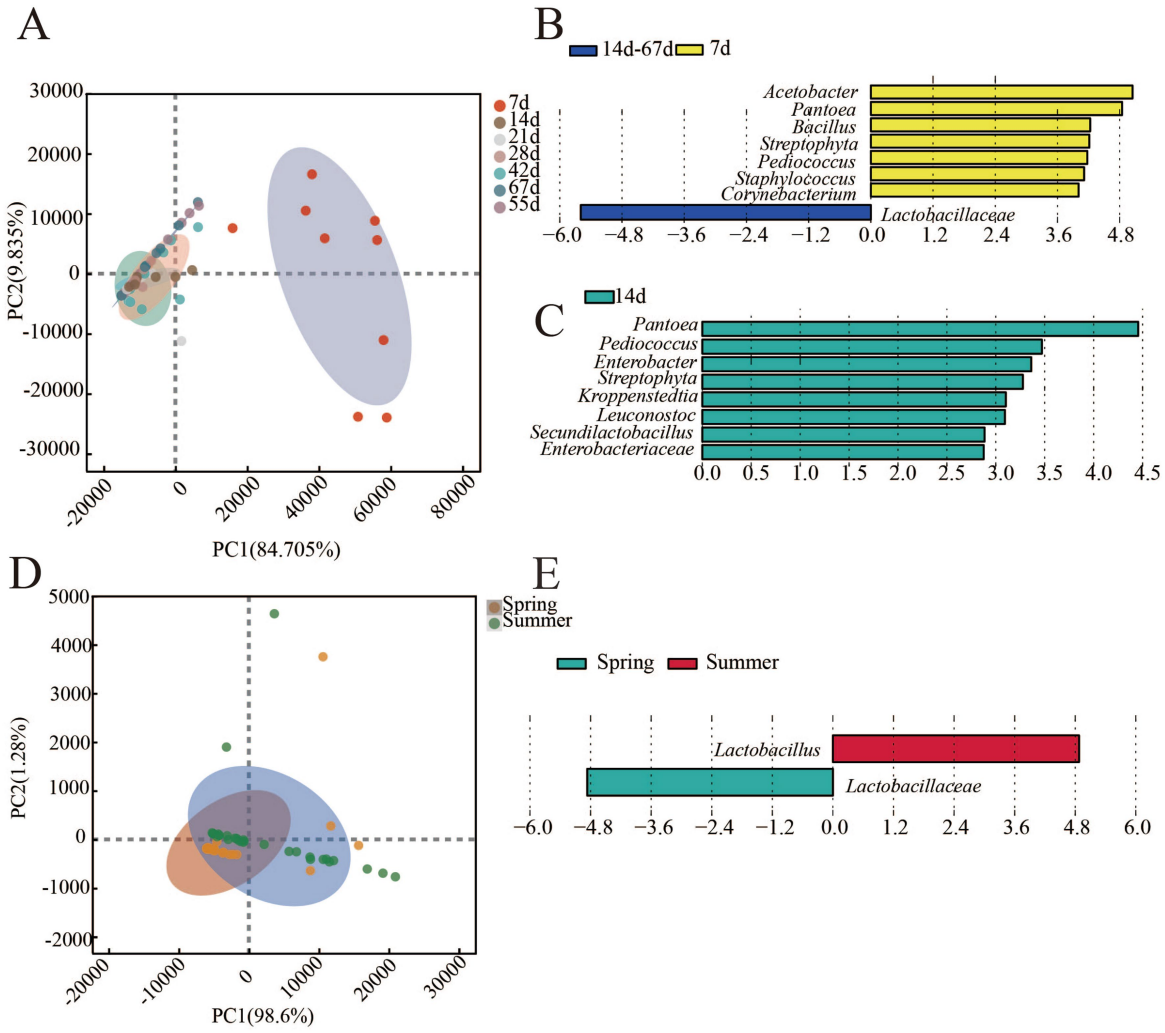
*β*-diversity and differential genus in FG of the fermentation process and different seasons (LDA>3, p<0.05). **(A)** β-diversity in FG of fermentation process. **(B)** Differential genus in FG of fermentation process. **(C)** Differential genus in FG of the lowest (14^th^ day) and highest (55^th^ day) concentration of AA. **(D)** β-diversity in FG of fermentation process. **(E)** Differential genus in FG of different seasons.

The bacterial community structure in different fermentation stage and in different seasons was different as shown in Figure 4. During the fermentation process, the results of PCA analysis showed that the distance between samples of 7th day and other time was relatively far (Figure 4A). It indicated that the bacterial community structure between 7th day and other time samples was different. The fermentation process could be divided into two stages (0–7 days and 14–67 days). Furthermore, LEfSe was applied to analyze differential genus between the two groups (Figure 4B) and the results showed that eight differential genera (LDA > 3, most of them was the dominant genus) were found between the early stage (0–7 day) and later stages (14–67 day) of fermentation process. *Acetobacter*, *Pantoea*, *Bacillus*, *Streptophyta*, *Pediococcus*, *Staphylococcus*, and *Corynebacterium* were differential genera of the early stage of fermentation, whereas *Lactobacillaceae* was the differential genus in the later stage of fermentation.

Furthermore, two groups of samples, the lowest (14th day) and highest (55th day) concentrations of AA, were selected to further analyze the differences in the bacterial community by LEfSe. The results (Figure 4C) showed that there were eight differential genera (*Pantoea*, *Pediococcus*, *Enterobacter*, *Streptophyta*, *Kroppenstedtia*, *Leuconostoc*, *Secundilactobacillus*, and *Enterobacteriaceae*) between the two groups, and these genera may be associated with AA synthesis during the fermentation process.

In different seasons, the structure of bacterial community also had differences (Figure 4D). *Lactobacillaceae* and *Lactobacillus* were identified as differential genera between spring and summer FG by LEfSe (Figure 4E). Although *Lactobacillaceae* and *Lactobacillus* have been identified as differential genus between summer and spring, the establishment of their association with AA synthesis requires further correlation analysis to elucidate the underlying relationship.

### 3.3 Correlation between AA, EA, bacteria, reducing sugar, acidity, and moisture in FG of the fermentation process and different seasons

#### 3.3.1 Correlation between AA and bacteria during the fermentation process and different seasons

Based on the Pearson correlation analysis, 305 genera of bacteria had significant negative correlation with AA during the fermentation process, and 54 genera of bacteria had significant positive (46) and negative (8) correlation with AA in different seasons (*p* < 0.05; Supplementary Tables S1; Supplementary Table S2). To find the closely related genera of AA, |R| > 0.5 and *p* < 0.05 were applied as the criteria to screen, and 21 genera were finally obtained (Table 1). The results showed that *Pantoea* was not only a differential genus between the high and low AA concentration sampling times during the fermentation process (Figure 4C), but also the abundance of *Pantoea* showed a significant negative correlation with AA (*p* < 0.05). However, the abundance of *Lactobacillaceae* and *Lactobacillus*, the differential genus between different seasons, showed no significant correlation (*p* > 0.05) with AA.

**Table 1:** Correlation between bacteria and AA.

No.	Genus	*R*	*p*
1	*Acidaminococcaceae*	0.51	0.000031
2	*Acinetobacter*	−0.52	0.000015
3	*unclassified_Bacilli*	0.56	0.000003
4	*Bacteroidetes*	0.53	0.000017
5	*Betaproteobacteria*	0.63	0.000000
6	*Brevibacterium*	−0.53	0.000006
7	*Brevundimonas*	−0.53	0.000007
8	*Comamonadaceae*	0.52	0.000023
9	*Facklamia*	−0.56	0.000006
10	*Fermentimonas*	0.55	0.000005
11	*Flavobacteriaceae*	0.56	0.000004
12	*Furfurilactobacillus*	−0.62	0.000006
13	*Lachnospiraceae*	0.61	0.000000
14	*Lactococcus*	−0.54	0.000006
15	*Pantoea*	−0.54	0.000006
16	*Raoultella*	−0.55	0.000006
17	*Saccharopolyspora*	−0.55	0.000006
18	*Sphaerochaeta*	0.53	0.000011
19	*Sporobacter*	0.57	0.000002
20	*Streptococcaceae*	0.52	0.000021
21	*Streptomycetaceae*	−0.53	0.000009

#### 3.3.2 Correlation of AA-related bacteria, reducing sugar, acidity, moisture, and dominant bacteria during the fermentation process and different seasons

**Figure 5:**
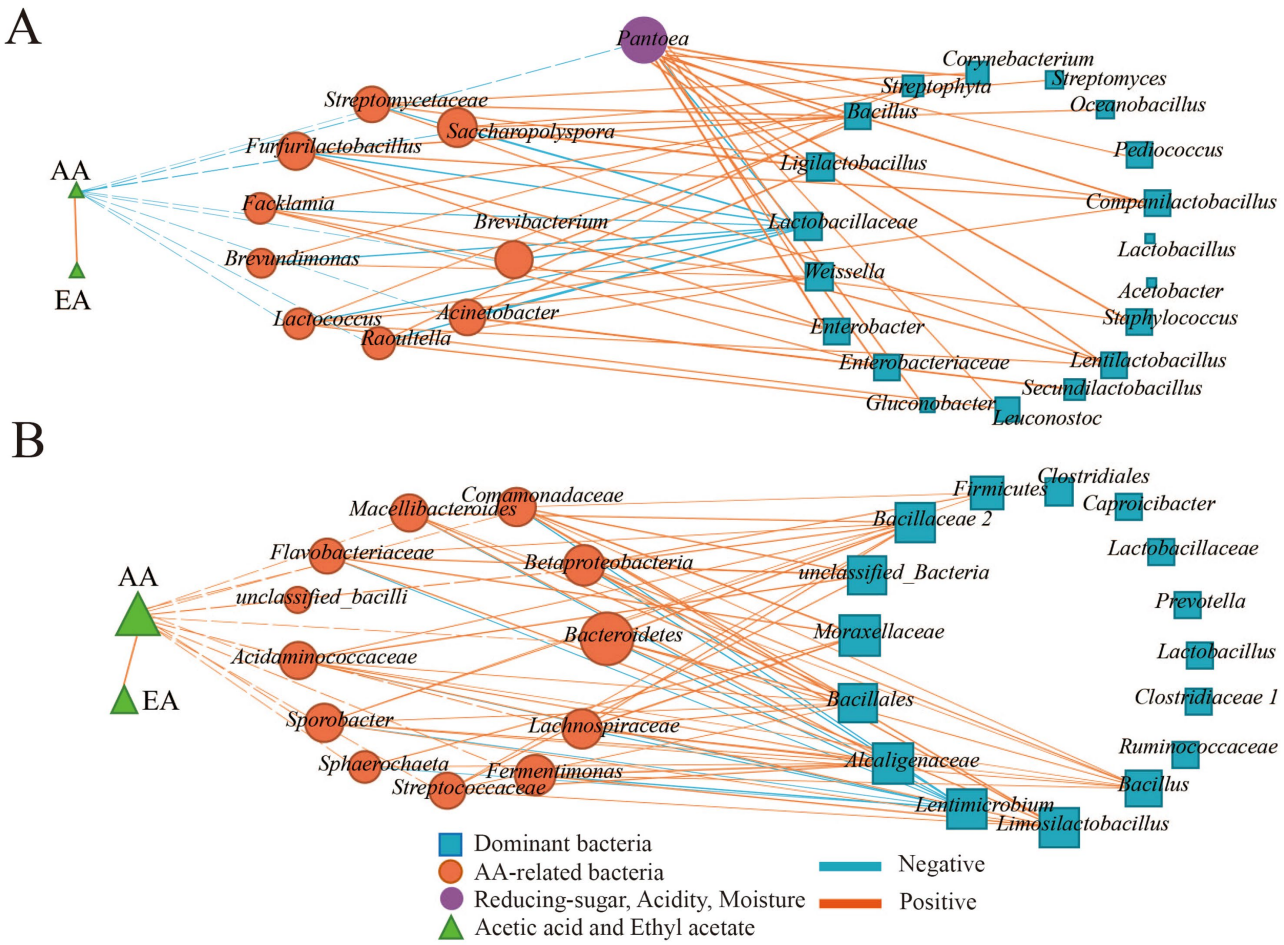
Correlation of AA-related bacteria, reducing sugar, acidity, moisture, and dominant bacteria of the fermentation process and different seasons. **(A)** Correlation of AA-related bacteria, reducing sugar, acidity, moisture and dominant bacteria in the fermentation process. **(B)** Correlation of AA-related bacteria, reducing sugar, acidity, moisture and dominant bacteria in different seasons.

During the fermentation process, environmental factors interacted with microorganisms and they were synergistically varied. To further understand the factors affecting AA-related bacteria, the relationships between AA-related bacteria, reducing sugar, acidity, moisture, and dominant bacteria during the fermentation process and different seasons were analyzed by Pearson correlation analysis (|R| > 0.5 and *p* < 0.05). As the results (Figure 5) showed EA was significantly correlated with AA (*p* < 0.05) during the fermentation process and across different seasons, indicating that the synthesis of EA was closely related to the concentration of AA during the fermentation process.

In this study, numerous bacteria were related to AA (Figure 5), and the relationship between the dominant genus, reducing sugar, acidity, moisture, and AA-related bacteria was further investigated. During the fermentation process (Figure 5A), significant correlations were observed among environmental factors. Acidity was positively correlated with both reducing sugar and moisture (*p* < 0.05), while reducing sugar had a negative correlation with moisture. However, no correlation was found between AA-related bacteria and environmental factors. There were 10 and 140 pairs of significant negative and positive correlations between AA-related bacteria and dominant bacteria. *Lactobacillaceae* was the main genus negatively related to AA-related bacteria (*p* < 0.05), while 16 dominant genera, such as *Weissella* had a positive correlation with AA-related bacteria (*p* < 0.05).

In different seasons (Figure 5B), moisture showed a positive correlation with acidity (*p* < 0.05) and a negative correlation with reducing sugar (*p* < 0.05). Additionally, AA-related bacteria such as *Fermentimonas*, *Bacteroidetes*, and *Betaproteobacteria* had a significant correlation with acidity and reducing sugar (*p* < 0.05). There were 12 and 96 pairs of significant negative and positive correlations between AA-related bacteria and dominant bacteria (*p* < 0.05). *Lentimicrobium* was the main genus negatively related to AA-related bacteria (*p* < 0.05), and nine dominant genera, such as *Moraxellaceae*, had a positive correlation with AA-related bacteria (*p* < 0.05).

### 3.4 Distribution characteristics of AA-related bacteria in RM, DQ^L^, DQ^F^, GE, PM, and TL

In the previous section of this study, AA-related bacteria and their influencing factors were investigated. However, there were various sources that contributed microorganisms to the fermentation, and understanding the distribution characteristics of AA-related bacteria in different sources was important to establish methods to regulate AA during the fermentation process. Based on the bacterial composition of the source (RM, DQ^L^, DQ^F^, GE, PM, and TL) (Figure 3C), the distribution characteristics of AA-related bacteria in different sources were analyzed in this section.

**Figure 6:**
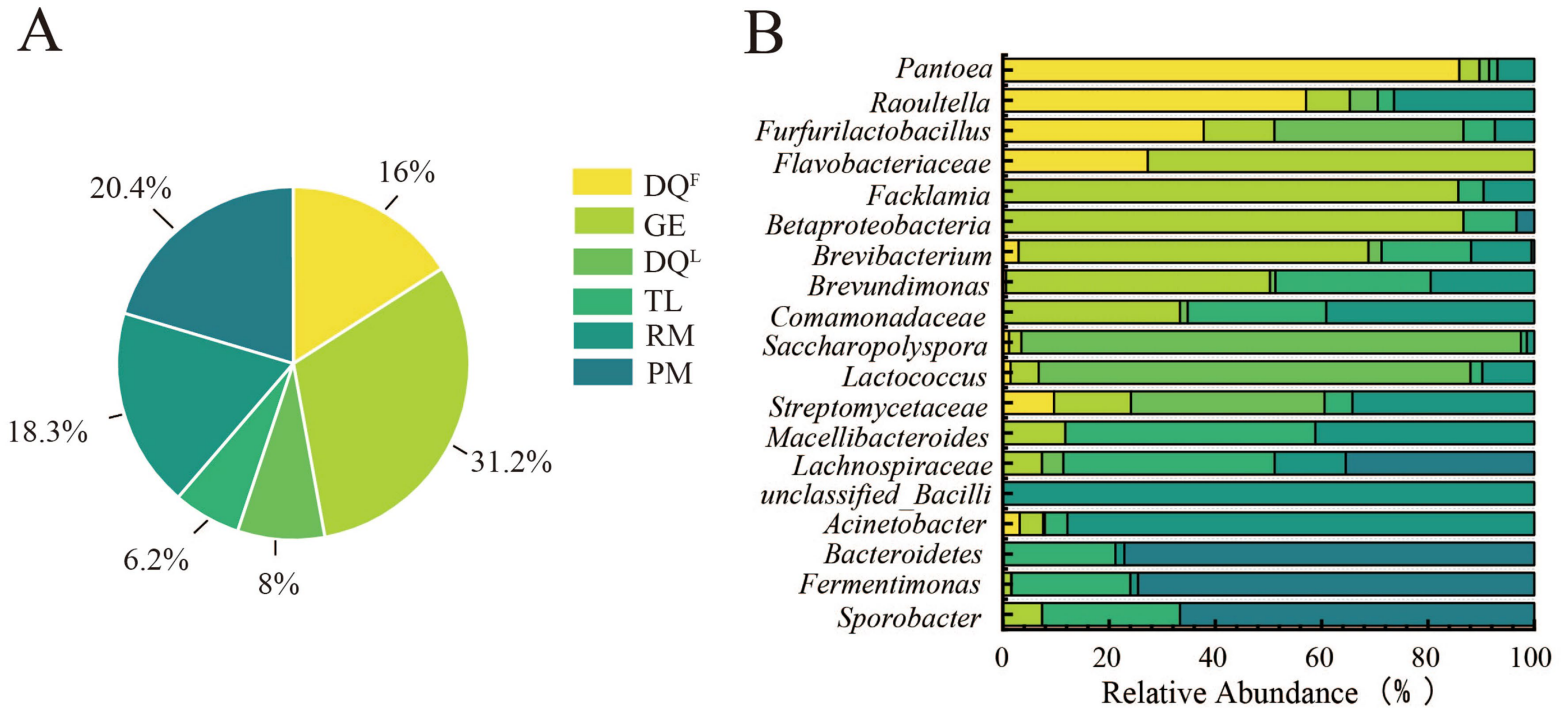
Distribution characteristics of AA-related bacteria in Raw Material (RM), Daqu (DQ^L^, DQ^F^), Pit Mud (PM), Ground (GE) and Tools (TL). **(A)** Contribution Ratio of Environmental Samples to Bacteria of FG. **(B)** Distribution of AA-associated bacterial genera in RM, DQ^L^, DQ^F^, GE, PM and TL.

Firstly, the sources of bacteria in 7th day FG were traced by FEAST and the results showed that the bacteria of 7th day FG were widely distributed in RM, DQ^L^, DQ^F^, GE, PM, and TL (Figure 6A). The bacteria in 7th day FG was mainly derived from GE (31.2%), TL (20.4%) and RM (18.3%). The starters, DQ^F^ and DQ^L^, contributed 16 and 8% of the bacteria to FG, while PM had the smallest contribution at 6.2%. In addition, the bacteria significantly related to AA (*p* < 0.05 and |R| > 0.5) had a complex distribution in RM, DQ^L^, DQ^F^, GE, PM, and TL. As the results showed (Figure 6B), RM could contribute *Acinetobacter* (88%), DQ^F^ and DQ^L^ could contribute *Pantoea* (86%) and *Saccharopolyspora* (94%), respectively. GE could contribute *Facklamia* (86%), *Brevibacteriu* (66%), and *Brevundimonas* (50%). However, PM and TL had a relatively low contribution of AA-related bacteria to the fermentation.

## 4 Discussion

The key flavor substances, ethyl caproate, ethyl lactate, ethyl acetate, and ethyl butyrate, in coordinated and balanced concentration, are important to keep the characteristics of SFB (Zou et al., 2018). The increase of AA concentration and the imbalance ratio of ethyl acetate can lead to a decrease in SFB quality. AA is the precursor of ethyl acetate, which is mainly produced by microbial metabolism during the fermentation process of Baijiu (Jiao et al., 2023; Li et al., 2017; Liu et al., 2023; Xie et al., 2022). Therefore, revealing the rules of AA synthesis during the fermentation of SFB, studying the AA-related microorganisms and their affecting factors, and tracing the microorganisms involved in AA synthesis are the basis for establishing methods to control AA during fermentation. Based on the above, the FG of SFB in northern China was sampled to study the changes of AA during the fermentation process and in different seasons. Moreover, the composition and distribution of AA-related bacteria in different sources (RM, DQ^L^, DQ^F,^ GE, PM, and TL) were revealed in this study.

As the results showed that AA and EA accumulated gradually during the fermentation process and there was a significant correlation between AA and EA (*p* < 0.05; Figure 5). In addition, AA and EA were significantly increased in summer FG and also had a significant correlation (*p* < 0.05; Figure 5). This indicated that AA accumulation had a significant effect on increase of EA. Low temperature in spring had a negative effect on AA accumulation than in summer and further affected EA accumulation (Zhang et al., 2016). It is reported that the synthesis of EA is closely related to AA (Wang L. et al., 2021; Xie et al., 2023; Xu Y. et al., 2022), and increasing the concentration of AA can enhance the concentration of EA in Light-flavor Baijiu (Li et al., 2022). EA can be synthesized by biological esterification during the fermentation process of Baijiu (Song et al., 2019).

In 67 days of fermentation, AA was mainly produced in the middle and later stages of fermentation (28–55 days, Figure 1A) and reached its highest concentration at 55 days of fermentation. In the early stage of fermentation (7–14 day), FG was rich in nutrients and air, bacteria could multiply rapidly and it led the high bacterial diversity and richness (Figure 2A). Moreover, acidity increased significantly at the beginning of fermentation (Figure 1C), but there was no significant accumulation of AA at this stage. It indicated that AA was not the reason for the increase in acidity. In addition, studies have shown that acids are produced by bacteria during the fermentation of Jian-flavor Baijiu, leading to an increase in acidity (Zhuansun et al., 2022). In this study, *Lactobacillus* gradually became the dominant genus in the middle and later stages of fermentation, and the acidity reached a maximum at 55 days of fermentation. High acidity could inhibit the growth of acid-intolerant bacteria (Zhang et al., 2005) and lead to the decline of diversity and richness of bacteria (Figure 2A).

During the fermentation process of Baijiu, the concentration of reducing sugar from the degradation of starch is an important factor for bacteria (Wu et al., 2023). In this study, the concentration of reducing sugar increased from 7 to 21 days of fermentation and gradually stabilized from 28 to 55 days of fermentation. The effects of acidity and alcohol accumulation on microorganisms are the main reason for the changes in reducing sugar in the later stages of fermentation (Zhuansun et al., 2022). Water is the medium for biochemical reactions, and the appropriate moisture can regulate the temperature in the cellar and reduce the acidity of the FG during the fermentation process (Zeng et al., 2024). In this study, the moisture of FG increased slowly throughout the fermentation process and gradually reached a stable level in the middle and later stages of fermentation.

In different seasons, the concentration of AA in summer FG was significantly higher than in spring (Figure 1B), which may be due to vigorous microbial growth and metabolism in summer caused by higher temperature (Zhang et al., 2016; Zhang et al., 2021). There were also significant differences in acidity and reducing sugar concentration in FG from different seasons (*p* < 0.05), a common phenomenon found in other related studies (Jiang et al., 2024; Guo et al., 2020). The average temperature in summer is higher than that in spring. Therefore, temperature change may be an important factor leading to the differences in bacterial diversity and AA content between spring and summer. SFB production in spring with lower temperatures has a positive effect on the content of AA. However, moisture had no significant difference between spring and summer FG, indicating that moisture was not a significant determinant of bacterial diversity and AA in this study (Chai et al., 2024). In addition, the acidity and reducing sugar of FG were higher in summer than in spring, which may also be attributed to the strong growth and metabolism of acid-producing bacteria, as well as saccharification caused by higher temperature (Zhang et al., 2021). The enhanced microbial metabolism during the summer at higher temperatures may be a contributing factor to the observed differences in acidity and reducing sugar concentration (Jiang et al., 2024). The higher diversity and richness of bacteria are found in summer FG (Figure 2B), and temperature may also be the main reason. A higher temperature has been observed to facilitate the growth of acid-producing bacteria, *Acetobacter* and *Lactobacillus* (De Filippis et al., 2018). It is commonly accepted that 35°C represents the optimal growth temperature for acid-producing bacteria, and seasonal variation had a significant influence on the diversity and richness of the bacterial community of FG (Torija et al., 2003).

Bacteria may be an important producer of AA, and understanding the bacterial community’s structural changes during the fermentation of SFB is important to reveal the synthetic law of AA. The enrichment of nutrients and a suitable environment allowed bacteria to multiply rapidly, and *Acetobacter* (22.1%), *Lactobacillaceae* (17.7%), *Pantoea* (12.9%), and *Lactobacillus* (10.8%) dominated the early stage of fermentation in this study. As fermentation progresses, the diversity and richness of bacteria gradually decrease in the later stages of fermentation due to the negative effects of acidity and ET. In the later stage of fermentation, *Lactobacillaceae* and *Lactobacillus* became the dominant genus and can increase the acidity by producing LA and AA to inhibit the growth of other microorganisms (He et al., 2022). Therefore, this may be the reason for the homogenization of bacterial genera with the fermentation process. Different seasons also affect the reproduction of microorganisms in FG (Sun et al., 2016) and the relative abundance of dominant genera, *Lactobacillaceae* and *Lactobacillus,* had a significant difference in different seasons. The temperature may be a factor that influences the bacterial community in SFB fermentation. In addition, some studies have shown that *Lactobacillaceae* and *Lactobacillus* were identified as key flavor-associated genera in Baijiu production and were responsible for the formation of a variety of flavor compounds such as AA, phenylacetic acid, and octanoic acid (Du et al., 2020; Hao et al., 2021; Xie et al., 2023). However, *Lactobacillaceae* and *Lactobacillus* had no significant correlation with AA (Table 1). During the fermentation process, AA accumulated gradually and stopped changing after 55 days of fermentation (Figure 1A); however, the bacterial community continued to change under the influence of other factors, which may have resulted in the above differences. In addition, studies showed that the fungal community of FG varied greatly at the end of fermentation in different seasons (Kang, 2022; Zhao et al., 2022), and fungi may be another factor influencing the AA concentration.

*Pantoea*, *Pediococcus*, *Enterobacter*, *Streptophyta*, *Kroppenstedtia*, *Leuconostoc*, *Secundilactobacillus*, and *Enterobacteriaceae* were the differential genera between the two time points (14th and 55th day) with significant difference in AA concentration during the fermentation process (Figures 1A, 4C). However, the differential genera in different seasons were *Lactobacillaceae* and *Lactobacillus*, although AA concentration showed significant differences between different seasons (Figures 1B, 4E). Therefore, it is difficult to clarify AA-related bacteria based on the perspective of differential genus, and correlation analysis was applied in this study to further search for the AA-related bacteria. As the results (Table 1) showed, 21 genera of bacteria related to AA were found in the two kinds of sampling methods. Eleven genera of bacteria had significant positive correlation (|R| > 0.5, *p* < 0.05) with AA (*Acidaminococcaceae*, *unclassified_Bacilli*, *Bacteroidetes*, *Betaproteobacteria*, *Comamonadaceae*, *Fermentimonas*, *Flavobacteriaceae*, *Lachnospiraceae*, *Sphaerochaeta*, *Sporobacter* and *Streptococcaceae*) and 10 genera (*Acinetobacter*, *Brevibacterium*, *Brevundimonas*, *Facklamia*, *Furfurilactobacillus*, *Lactococcus*, *Pantoea*, *Raoultella*, *Saccharopolyspora*, and *Streptomycetaceae*) showed significant negative correlation (|R| > 0.5, *p* < 0.05) with AA. Based on the results of composition analysis, differential analysis, and correlation analysis, *Pantoea* was the dominant, AA-related, and differential genus during the fermentation process. This suggests that *Pantoea* may be a key genus for affecting AA during the fermentation process of SFB. Moreover, the results of the correlation analysis (Figure 5) showed that EA was significantly correlated with AA. Similarly, the genus of AA-related bacteria was significantly correlated with the dominant genera of bacteria (*p* < 0.05). The succession of FG bacteria was driven by physicochemical factors, and the changes of AA were the result of the synergistic effect of physicochemical factors and microorganism succession.

The microorganisms involved during the fermentation process of Baijiu come from different sources (Zhang et al., 2005), and tracing the sources of AA-related microorganisms is important to build a regulating method of AA during the fermentation process. In this study, the bacterial composition of the microbial source (RM, DQ^L^, DQ^F^, GE, PM, and TL) was analyzed, and the distribution of AA-related bacteria was investigated. The results showed that RM contributes mainly to *Acinetobacter* and *unclassified_Bacilli*. *Unclassified_Bacilli* had the ability of hydrolysis and high-temperature resistance (Beaumont, 2002), and this was consistent with the fact that RM had been steamed during the fermentation process of Baijiu.

Daqu is considered the main source of microorganisms for Baijiu fermentation (He et al., 2022). However, DQ^F^ and DQ^L^ contributed 16 and 8% of the bacteria in this study. From the perspective of AA-related bacteria, DQ^F^ mainly contributed the genera of *Pantoea*, *Raoultella*, and *Furfurilactobacillus*, and DQ^L^ mainly contributed the genera of *Saccharopolyspora*, *Lactococcus*, and *Streptococcaceae*. It is reported that *Pantoea* widely presents medium and high-temperature Daqu, but its function in Baijiu fermentation is unclear (Gou et al., 2015). In this study, *Pantoea* was the dominant genus in DQ^L^ and showed a significant negative correlation with AA (*p* < 0.05) during the fermentation process. All of those indicated that *Pantoea* and AA had a close relationship during the fermentation process. *Saccharopolyspora*, the dominant bacteria in DQ, is crucial for the production of flavor substances in Baijiu (Gan et al., 2019) and was significantly negatively correlated with AA in this study (*p* < 0.05). *Lactococcus,* a common genus in Baijiu-related studies (Zheng et al., 2015), was a dominant genus in DQ^L^ and DQ^F^, and showed a significant negative correlation (*p* < 0.05) with AA in this study.

The microorganism in the brewing environment was the primary source of microorganisms in FG, except for DQ and RM (Hao et al., 2021; Qian et al., 2021b; Rintala et al., 2008). During the fermentation process of Baijiu, most of the manipulation proceeded under the ground (GE), and it may be the main source of microorganisms for FG (Zhang, 2023). But in the prospect of AA-related bacteria, GE contributed the low relative abundance genera such as *Facklamia*, *Flavobacteriaceae*, *Betaproteobacteria*, *Brevibacteriu*, and *Brevundimonas*. *Facklamia* was the common genus in building and house dust (Rintala et al., 2008), *Flavobacteriaceae* and *Betaproteobacteria* were the dominant bacteria in the environment of the Bajiu fermentation process (Wang Q. et al., 2021). *Brevibacteriu* and *Brevundimonas* were common genus of soil (Naqqash et al., 2020). However, the function of the above low-abundance AA-related bacteria in Baijiu fermentation was unknown. The relative abundance of AA-related bacteria in PM and TL was lower than in other environmental samples. It is reported that microorganisms in PM cannot infiltrate excessively into FG at the early stage of fermentation, and PM mainly acts as the main source of bacteria at the end of fermentation (Liang et al., 2015; Qian et al., 2021).

AA-related bacteria were distributed in the sources of RM, DQ^L^, DQ^F^, GE, PM, and TL in different proportions. The suggestions for regulating AA during the fermentation process were proposed based on the above results in this study. DQ^F^ could provide three genera of bacteria that negatively correlated with AA (*p* < 0.05), and their relative abundance order was *Pantoea* > *Raoultella* > *Furfurilactobacillus*. In addition, *Pantoea* was the dominant genus during the fermentation process. Therefore, DQ^F^ can be applied to inhibit the increase of AA by enhancing the inoculation proportion of DQ^F^ during the fermentation process. Although DQ^L^ can provide genera with AA-negative correlation (*p* < 0.05) (*Saccharopolyspora*, *Lactococcus*, and *Streptomycetaceae*), they were not the dominant genus during the fermentation process. Replacing different types of PM may also have a positive effect on reducing AA, because PM can provide some low-abundance AA-related bacteria, such as *Bacteroidetes*, *Fermentimonas*, Sporobact*er*, and *Comamonadacede*. *Unclassified_Bacilli* in FG had a positive relation with AA and mainly came from RM. The function of *Unclassified_Bacilli* is to hydrolyze starch (Beaumont, 2002) and RM cannot be replaced by others in the Baijiu fermentation. Therefore, replacing RM is not suitable for regulating AA during the fermentation process. The relative abundance of AA-related genera in GE and TL was lower than in other environmental samples. Although they can provide *Betaproteobacteria, Facklamia, Flavobacteriaceae, Brevibacterium, Brevundimonas, Lachnospiraceae*, and *Sporobacter,* they are hard to operate in regulating AA during the fermentation process.

However, correlation analysis under non-cultural conditions has certain limitations. The results of the correlation analysis cannot establish a direct causal relationship between the microorganisms and AA. In the next step of the study, isolation of AA-related microorganisms by culture techniques will be carried out to further reveal the regulation of AA synthesis in SFB.

## 5 Conclusion

Traditional fermented foods are mainly produced openly, and various microorganisms can participate during the fermentation process. Clarification of key flavor substance-related microorganisms is the basis for understanding the mechanism of flavor formation in traditional fermented foods, and elucidation of the distribution characteristics of key flavor substance-related microorganisms is important to establish regulation methods of key flavor substances. In the production process of SFB, “increasing ethyl caproate and decreasing EA” are important to improve the quality of products. In this study, the concentration of AA increased during the fermentation process, and AA in summer was significantly higher than in spring (*p* < 0.05). A total of 23 genera were significantly related to AA (*p* < 0.05), and most of them were also significantly related (*p* < 0.05) to the dominant genus, reducing sugar, moisture, and acidity. Moreover, *Pantoea* (negatively related to AA) mainly comes from Daqu^F^ and was the dominant genus during the fermentation process. *Saccharopolyspora, Lactococcus*, *and Streptomycetaceae,* the low-abundance negatively related bacteria, mainly originated from Daqu^L^ and pit mud. *Unclassified_Bacilli* had a positive relationship with AA and mainly came from raw material. Ground and tools can provide seven genera by low relative abundance. This study provided a theoretical reference for controlling AA synthesis in aspects of the selection of fermentation time and Daqu in SFB production.

## Data Availability

The original contributions presented in the study are publicly available. This data can be found here: [https://www.ncbi.nlm.nih.gov/sra/PRJNA1286820; Aceesion number: PRJNA1286820].

## Footnotes

^1^ http://www.mothur.org

^2^ https://github.com/cozygene/FEAST
