# Supplementary material for: Composition and distribution characteristics of acetic acid-related bacteria during the fermentation process of strong-flavor baijiu
Source: Front Microbiol. 2025 Jul 17;16:1603412. doi: 10.3389/fmicb.2025.1603412 (PMC12310668; doi:10.3389/fmicb.2025.1603412)
Supplement: Supplementary file 1 [file Table_1.docx]

Supplementary Material

**Table S1** Correlation between bacteria and AA of fermentation process.

| No | Genus | R | p |
| --- | --- | --- | --- |
| 1 | *Furfurilactobacillus* | -0.62 | 0.00001 |
| 2 | *Facklamia* | -0.56 | 0.00001 |
| 3 | *Raoultella* | -0.55 | 0.00001 |
| 4 | *Saccharopolyspora* | -0.55 | 0.00001 |
| 5 | *Pantoea* | -0.54 | 0.00001 |
| 6 | *Lactococcus* | -0.54 | 0.00001 |
| 7 | *Brevibacterium* | -0.53 | 0.00001 |
| 8 | *Brevundimonas* | -0.53 | 0.00001 |
| 9 | *Streptomycetaceae* | -0.53 | 0.00001 |
| 10 | *Acinetobacter* | -0.52 | 0.00002 |
| 11 | *Solirubrobacterales* | -0.51 | 0.00002 |
| 12 | *Liquorilactobacillus* | -0.50 | 0.00003 |
| 13 | *Myroides* | -0.50 | 0.00003 |
| 14 | *Mycobacterium* | -0.49 | 0.00004 |
| 15 | *Sedimentibacter* | -0.49 | 0.00004 |
| 16 | *Kroppenstedtia* | -0.49 | 0.00004 |
| 17 | *Ralstonia* | -0.49 | 0.00004 |
| 18 | *Gemmatimonadetes* | -0.49 | 0.00005 |
| 19 | *Rhizobiales* | -0.48 | 0.00006 |
| 20 | *Sphingobacteriaceae* | -0.48 | 0.00007 |
| 21 | *Streptophyta* | -0.48 | 0.00007 |
| 22 | *Gaiella* | -0.48 | 0.00008 |
| 23 | *Latilactobacillus* | -0.47 | 0.00009 |
| 24 | *Bradyrhizobium* | -0.47 | 0.00009 |
| 25 | *unclassified_Betaproteobacteria* | -0.47 | 0.00010 |
| 26 | *Desemzia* | -0.47 | 0.00010 |
| 27 | *Kocuria* | -0.47 | 0.00011 |
| 28 | *Loigolactobacillus* | -0.47 | 0.00011 |
| 29 | *Lactiplantibacillus* | -0.47 | 0.00011 |
| 30 | *Mycetohabitans* | -0.47 | 0.00012 |
| 31 | *Terrisporobacter* | -0.46 | 0.00012 |
| 32 | *Sporolactobacillus* | -0.46 | 0.00014 |
| 33 | *Paracoccus* | -0.46 | 0.00018 |
| 34 | *unclassified_Eggerthellaceae* | -0.45 | 0.00018 |
| 35 | *Serratia* | -0.45 | 0.00018 |
| 36 | *unclassified_Deltaproteobacteria* | -0.45 | 0.00018 |
| 37 | *Glutamicibacter* | -0.45 | 0.00019 |
| 38 | *unclassified_Muribaculaceae* | -0.45 | 0.00019 |
| 39 | *Sphingomonas* | -0.45 | 0.00020 |
| 40 | *Aquamicrobium* | -0.45 | 0.00020 |
| 41 | *unclassified_Enterobacteriaceae* | -0.45 | 0.00021 |
| 42 | *Phenylobacterium* | -0.45 | 0.00022 |
| 43 | *Clostridium sensu stricto* | -0.45 | 0.00024 |
| 44 | *unclassified_Clostridiaceae* | -0.45 | 0.00025 |
| 45 | *Leuconostoc* | -0.44 | 0.00034 |
| 46 | *Advenella* | -0.44 | 0.00035 |
| 47 | *Acidovorax* | -0.43 | 0.00037 |
| 48 | *Yersinia* | -0.43 | 0.00037 |
| 49 | *unclassified_Enterobacterales* | -0.43 | 0.00037 |
| 50 | *unclassified_Acetobacteraceae* | -0.43 | 0.00039 |
| 51 | *unclassified_Micrococcales* | -0.43 | 0.00039 |
| 52 | *unclassified_Erwiniaceae* | -0.43 | 0.00042 |
| 53 | *unclassified_Actinobacteria* | -0.43 | 0.00044 |
| 54 | *Alcaligenes* | -0.43 | 0.00044 |
| 55 | *Gluconobacter* | -0.43 | 0.00046 |
| 56 | *Gelidibacter* | -0.43 | 0.00049 |
| 57 | *Sphingobacterium* | -0.43 | 0.00049 |
| 58 | *unclassified_Dysgonomonadaceae* | -0.42 | 0.00052 |
| 59 | *Gp6* | -0.42 | 0.00052 |
| 60 | *Devosia* | -0.42 | 0.00053 |
| 61 | *Limosilactobacillus* | -0.42 | 0.00053 |
| 62 | *unclassified_Planctomycetaceae* | -0.42 | 0.00055 |
| 63 | *Arboricoccus* | -0.42 | 0.00059 |
| 64 | *Vagococcus* | -0.42 | 0.00060 |
| 65 | *Luteococcus* | -0.42 | 0.00060 |
| 66 | *unclassified_Bacillaceae 2* | -0.42 | 0.00067 |
| 67 | *Lysobacter* | -0.42 | 0.00070 |
| 68 | *unclassified_Thermoleophilia* | -0.42 | 0.00071 |
| 69 | *unclassified_Clostridiales* | -0.41 | 0.00074 |
| 70 | *Nocardioides* | -0.41 | 0.00079 |
| 71 | *Gp25* | -0.41 | 0.00082 |
| 72 | *Sporacetigenium* | -0.41 | 0.00087 |
| 73 | *Psychrobacter* | -0.41 | 0.00088 |
| 74 | *unclassified_Chloroplast* | -0.41 | 0.00089 |
| 75 | *Pediococcus* | -0.41 | 0.00092 |
| 76 | *unclassified_Burkholderiales* | -0.41 | 0.00093 |
| 77 | *Nesterenkonia* | -0.40 | 0.00101 |
| 78 | *Paenochrobactrum* | -0.40 | 0.00101 |
| 79 | *Rhodococcus* | -0.40 | 0.00119 |
| 80 | *unclassified_Carnobacteriaceae* | -0.40 | 0.00123 |
| 81 | *unclassified_Proteobacteria* | -0.40 | 0.00125 |
| 82 | *Shinella* | -0.40 | 0.00128 |
| 83 | *unclassified_Sphaerobacteraceae* | -0.40 | 0.00130 |
| 84 | *Atlantibacter* | -0.39 | 0.00138 |
| 85 | *unclassified_Rhodospirillales* | -0.39 | 0.00139 |
| 86 | *Alkalibaculum* | -0.39 | 0.00149 |
| 87 | *unclassified_Acidimicrobiales* | -0.39 | 0.00152 |
| 88 | *unclassified_Bacteria* | -0.39 | 0.00158 |
| 89 | *Ligilactobacillus* | -0.39 | 0.00159 |
| 90 | *Providencia* | -0.39 | 0.00160 |
| 91 | *Empedobacter* | -0.39 | 0.00165 |
| 92 | *Bifidobacterium* | -0.39 | 0.00167 |
| 93 | *Bordetella* | -0.39 | 0.00167 |
| 94 | *unclassified_Microbacteriaceae* | -0.39 | 0.00168 |
| 95 | *unclassified_Azospirillaceae* | -0.39 | 0.00169 |
| 96 | *unclassified_Bacilli* | -0.39 | 0.00171 |
| 97 | *unclassified_Lachnospiraceae* | -0.39 | 0.00172 |
| 98 | *Corynebacterium* | -0.39 | 0.00178 |
| 99 | *unclassified_Gemmatimonadaceae* | -0.38 | 0.00184 |
| 100 | *Rhizobium* | -0.38 | 0.00185 |
| 101 | *Amylolactobacillus* | -0.38 | 0.00193 |
| 102 | *unclassified_Bifidobacteriaceae* | -0.38 | 0.00195 |
| 103 | *Gp4* | -0.38 | 0.00203 |
| 104 | *unclassified_Iamiaceae* | -0.38 | 0.00210 |
| 105 | *Marmoricola* | -0.38 | 0.00210 |
| 106 | *Dietzia* | -0.38 | 0.00211 |
| 107 | *Patulibacter* | -0.38 | 0.00219 |
| 108 | *unclassified_Planococcaceae* | -0.38 | 0.00223 |
| 109 | *Aneurinibacillus* | -0.38 | 0.00223 |
| 110 | *Moheibacter* | -0.38 | 0.00223 |
| 111 | *Planctomicrobium* | -0.38 | 0.00228 |
| 112 | *Virgibacillus* | -0.38 | 0.00232 |
| 113 | *Gp7* | -0.38 | 0.00237 |
| 114 | *Anaeromyxobacter* | -0.38 | 0.00238 |
| 115 | *unclassified_Cytophagales* | -0.38 | 0.00245 |
| 116 | *Stenotrophobacter* | -0.37 | 0.00259 |
| 117 | *Lysinibacillus* | -0.37 | 0.00273 |
| 118 | *Caproicibacter* | -0.37 | 0.00275 |
| 119 | *Bavariicoccus* | -0.37 | 0.00278 |
| 120 | *Staphylococcus* | -0.37 | 0.00284 |
| 121 | *Pseudonocardia* | -0.37 | 0.00286 |
| 122 | *Enhydrobacter* | -0.37 | 0.00286 |
| 123 | *Paludisphaera* | -0.37 | 0.00307 |
| 124 | *Deinococcus* | -0.37 | 0.00308 |
| 125 | *Gp16* | -0.37 | 0.00310 |
| 126 | *Oligella* | -0.37 | 0.00321 |
| 127 | *unclassified_Rhodobacteraceae* | -0.36 | 0.00336 |
| 128 | *Clostridium XlVa* | -0.36 | 0.00349 |
| 129 | *Leucobacter* | -0.36 | 0.00355 |
| 130 | *unclassified_Ruminococcaceae* | -0.36 | 0.00355 |
| 131 | *Anaerocolumna* | -0.36 | 0.00356 |
| 132 | *Reyranella* | -0.36 | 0.00363 |
| 133 | *Lacrimispora* | -0.36 | 0.00365 |
| 134 | *Solibacillus* | -0.36 | 0.00367 |
| 135 | *Lentibacillus* | -0.36 | 0.00375 |
| 136 | *Polynucleobacter* | -0.36 | 0.00380 |
| 137 | *Secundilactobacillus* | -0.36 | 0.00380 |
| 138 | *Acidipropionibacterium* | -0.36 | 0.00385 |
| 139 | *Brucella* | -0.36 | 0.00386 |
| 140 | *Solirubrobacter* | -0.36 | 0.00386 |
| 141 | *Companilactobacillus* | -0.36 | 0.00395 |
| 142 | *Agromyces* | -0.36 | 0.00401 |
| 143 | *Phocaeicola* | -0.36 | 0.00406 |
| 144 | *unclassified_Chloroflexi* | -0.36 | 0.00410 |
| 145 | *unclassified_Eubacteriaceae* | -0.36 | 0.00416 |
| 146 | *Pelagibacterium* | -0.36 | 0.00429 |
| 147 | *Ammoniphilus* | -0.35 | 0.00434 |
| 148 | *Mobilicoccus* | -0.35 | 0.00437 |
| 149 | *Nitrosomonas* | -0.35 | 0.00444 |
| 150 | *unclassified_Methylophilaceae* | -0.35 | 0.00460 |
| 151 | *Pseudomonas* | -0.35 | 0.00466 |
| 152 | *Chryseobacterium* | -0.35 | 0.00468 |
| 153 | *unclassified_Planctomycetacia* | -0.35 | 0.00468 |
| 154 | *Georgenia* | -0.35 | 0.00479 |
| 155 | *Sphingorhabdus* | -0.35 | 0.00489 |
| 156 | *Flaviflexus* | -0.35 | 0.00502 |
| 157 | *Microvirga* | -0.35 | 0.00503 |
| 158 | *Hyphomicrobium* | -0.35 | 0.00504 |
| 159 | *Komagataeibacter* | -0.35 | 0.00510 |
| 160 | *Schleiferilactobacillus* | -0.35 | 0.00523 |
| 161 | *Ignatzschineria* | -0.35 | 0.00525 |
| 162 | *unclassified_Pirellulales* | -0.35 | 0.00526 |
| 163 | *Baekduia* | -0.35 | 0.00543 |
| 164 | *Tissierella* | -0.35 | 0.00555 |
| 165 | *unclassified_Gammaproteobacteria* | -0.35 | 0.00560 |
| 166 | *Sediminibacterium* | -0.34 | 0.00572 |
| 167 | *Hazenella* | -0.34 | 0.00577 |
| 168 | *Helcococcus* | -0.34 | 0.00587 |
| 169 | *Gp22* | -0.34 | 0.00597 |
| 170 | *Blastococcus* | -0.34 | 0.00607 |
| 171 | *Gracilibacillus* | -0.34 | 0.00610 |
| 172 | *Aquihabitans* | -0.34 | 0.00624 |
| 173 | *Microbacterium* | -0.34 | 0.00658 |
| 174 | *Escherichia/Shigella* | -0.34 | 0.00667 |
| 175 | *Streptococcus* | -0.34 | 0.00677 |
| 176 | *unclassified_Xanthomonadales* | -0.34 | 0.00678 |
| 177 | *unclassified_Geminicoccaceae* | -0.34 | 0.00688 |
| 178 | *Atopostipes* | -0.34 | 0.00699 |
| 179 | *unclassified_Intrasporangiaceae* | -0.34 | 0.00707 |
| 180 | *Ureibacillus* | -0.33 | 0.00732 |
| 181 | *Comamonas* | -0.33 | 0.00734 |
| 182 | *Candidatus Pelagibacter* | -0.33 | 0.00738 |
| 183 | *Aeromicrobium* | -0.33 | 0.00742 |
| 184 | *Saccharibacteria _incertae_sedis* | -0.33 | 0.00742 |
| 185 | *Ilumatobacter* | -0.33 | 0.00746 |
| 186 | *Gp10* | -0.33 | 0.00762 |
| 187 | *Bosea* | -0.33 | 0.00775 |
| 188 | *Stenotrophomonas* | -0.33 | 0.00783 |
| 189 | *Daejeonia* | -0.33 | 0.00793 |
| 190 | *Fimbriiglobus* | -0.33 | 0.00821 |
| 191 | *Hydrogenispora* | -0.33 | 0.00864 |
| 192 | *Sphingobium* | -0.33 | 0.00901 |
| 193 | *Risungbinella* | -0.33 | 0.00904 |
| 194 | *Bhargavaea* | -0.33 | 0.00910 |
| 195 | *Paucilactobacillus* | -0.33 | 0.00916 |
| 196 | *unclassified_Myxococcales* | -0.33 | 0.00926 |
| 197 | *Algisphaera* | -0.32 | 0.00959 |
| 198 | *unclassified_Steroidobacteraceae* | -0.32 | 0.00962 |
| 199 | *Enterobacter* | -0.32 | 0.01017 |
| 200 | *Sporosarcina* | -0.32 | 0.01043 |
| 201 | *Paramuribaculum* | -0.32 | 0.01048 |
| 202 | *Turicibacter* | -0.32 | 0.01108 |
| 203 | *Luteimonas* | -0.32 | 0.01114 |
| 204 | *Actinomarinicola* | -0.32 | 0.01122 |
| 205 | *Enhygromyxa* | -0.32 | 0.01139 |
| 206 | *Brachybacterium* | -0.32 | 0.01141 |
| 207 | *Pseudarcobacter* | -0.32 | 0.01155 |
| 208 | *Arenimonas* | -0.32 | 0.01167 |
| 209 | *unclassified_Sphingomonadales* | -0.32 | 0.01191 |
| 210 | *Sphingopyxis* | -0.31 | 0.01217 |
| 211 | *unclassified_Isosphaeraceae* | -0.31 | 0.01312 |
| 212 | *Olsenella* | -0.31 | 0.01313 |
| 213 | *Duncaniella* | -0.31 | 0.01339 |
| 214 | *Glycomyces* | -0.31 | 0.01342 |
| 215 | *Gordonia* | -0.31 | 0.01362 |
| 216 | *Kofleria* | -0.31 | 0.01414 |
| 217 | *Weissella* | -0.31 | 0.01445 |
| 218 | *unclassified_Phyllobacteriaceae* | -0.31 | 0.01463 |
| 219 | *Ornithinimicrobium* | -0.30 | 0.01510 |
| 220 | *unclassified_Thermoguttaceae* | -0.30 | 0.01524 |
| 221 | *unclassified_Comamonadaceae* | -0.30 | 0.01530 |
| 222 | *Stackebrandtia* | -0.30 | 0.01588 |
| 223 | *Paeniclostridium* | -0.30 | 0.01616 |
| 224 | *Methylocystis* | -0.30 | 0.01642 |
| 225 | *Vulgatibacter* | -0.30 | 0.01642 |
| 226 | *Odoribacter* | -0.30 | 0.01688 |
| 227 | *Spartobacteria_genera_incertae_sedis* | -0.30 | 0.01707 |
| 228 | *Flavobacterium* | -0.30 | 0.01708 |
| 229 | *Pusillimonas* | -0.30 | 0.01729 |
| 230 | *Marinilactibacillus* | -0.30 | 0.01732 |
| 231 | *Prauserella* | -0.30 | 0.01755 |
| 232 | *Parapedobacter* | -0.30 | 0.01812 |
| 233 | *Pseudochelatococcus* | -0.30 | 0.01835 |
| 234 | *Lapidilactobacillus* | -0.30 | 0.01885 |
| 235 | *Luteolibacter* | -0.29 | 0.01898 |
| 236 | *unclassified_Xanthomonadaceae* | -0.29 | 0.01966 |
| 237 | *Phreatobacter* | -0.29 | 0.01971 |
| 238 | *unclassified_Flavobacteriaceae* | -0.29 | 0.02008 |
| 239 | *Pirellula* | -0.29 | 0.02033 |
| 240 | *Propionibacterium* | -0.29 | 0.02039 |
| 241 | *unclassified_Nannocystaceae* | -0.29 | 0.02040 |
| 242 | *Rhabdobacter* | -0.29 | 0.02099 |
| 243 | *unclassified_Alcaligenaceae* | -0.29 | 0.02142 |
| 244 | *Romboutsia* | -0.29 | 0.02254 |
| 245 | *Tepidisphaera* | -0.29 | 0.02283 |
| 246 | *Anaerosalibacter* | -0.29 | 0.02313 |
| 247 | *unclassified_Lactobacillales* | -0.29 | 0.02336 |
| 248 | *Pseudolabrys* | -0.28 | 0.02398 |
| 249 | *Proteiniclasticum* | -0.28 | 0.02451 |
| 250 | *Aquisphaera* | -0.28 | 0.02456 |
| 251 | *unclassified_Bacillaceae 1* | -0.28 | 0.02502 |
| 252 | *Gemmatimonas* | -0.28 | 0.02574 |
| 253 | *unclassified_Planctomycetales* | -0.28 | 0.02586 |
| 254 | *WPS-1_genera_incertae_sedis* | -0.28 | 0.02608 |
| 255 | *Prevotellamassilia* | -0.28 | 0.02619 |
| 256 | *Schaalia* | -0.28 | 0.02712 |
| 257 | *Jeotgalicoccus* | -0.28 | 0.02788 |
| 258 | *Rhabdanaerobium* | -0.28 | 0.02790 |
| 259 | *Rummeliibacillus* | -0.28 | 0.02791 |
| 260 | *Schnuerera* | -0.28 | 0.02799 |
| 261 | *Camelimonas* | -0.28 | 0.02806 |
| 262 | *Truepera* | -0.28 | 0.02819 |
| 263 | *Proteus* | -0.28 | 0.02862 |
| 264 | *Clostridioides* | -0.27 | 0.02925 |
| 265 | *Parcubacteria_genera_incertae_sedis* | -0.27 | 0.02944 |
| 266 | *Desertimonas* | -0.27 | 0.03073 |
| 267 | *Lentilactobacillus* | -0.27 | 0.03078 |
| 268 | *Lujinxingia* | -0.27 | 0.03082 |
| 269 | *unclassified_Yersiniaceae* | -0.27 | 0.03132 |
| 270 | *Aurantimonas* | -0.27 | 0.03139 |
| 271 | *Enterococcus* | -0.27 | 0.03199 |
| 272 | *Pelomonas* | -0.27 | 0.03223 |
| 273 | *unclassified_Oxalobacteraceae* | -0.27 | 0.03223 |
| 274 | *Cereibacter* | -0.27 | 0.03239 |
| 275 | *Pseudofulvimonas* | -0.27 | 0.03286 |
| 276 | *Macellibacteroides* | -0.27 | 0.03293 |
| 277 | *Cerasibacillus* | -0.27 | 0.03361 |
| 278 | *Erythrobacter* | -0.27 | 0.03362 |
| 279 | *Stappia* | -0.27 | 0.03379 |
| 280 | *Acetobacter* | -0.27 | 0.03382 |
| 281 | *Steroidobacter* | -0.27 | 0.03426 |
| 282 | *unclassified_Ilumatobacteraceae* | -0.27 | 0.03545 |
| 283 | *Erysipelothrix* | -0.27 | 0.03578 |
| 284 | *Brochothrix* | -0.26 | 0.03661 |
| 285 | *Mesorhizobium* | -0.26 | 0.03689 |
| 286 | *Halotalea* | -0.26 | 0.03739 |
| 287 | *unclassified_Gemmataceae* | -0.26 | 0.03808 |
| 288 | *Pelagibius* | -0.26 | 0.03849 |
| 289 | *Acidibacter* | -0.26 | 0.03878 |
| 290 | *Roseimaritima* | -0.26 | 0.03890 |
| 291 | *Sebaldella* | -0.26 | 0.03904 |
| 292 | *Limnobacter* | -0.26 | 0.03906 |
| 293 | *unclassified_Hyphomicrobiaceae* | -0.26 | 0.03924 |
| 294 | *Saccharomonospora* | -0.26 | 0.04096 |
| 295 | *unclassified_Thermoactinomycetaceae* | -0.26 | 0.04096 |
| 296 | *unclassified_Alphaproteobacteria* | -0.26 | 0.04127 |
| 297 | *Gp5* | -0.26 | 0.04249 |
| 298 | *Piscicoccus* | -0.26 | 0.04266 |
| 299 | *Peribacillus* | -0.26 | 0.04325 |
| 300 | *Cohnella* | -0.25 | 0.04420 |
| 301 | *Novosphingobium* | -0.25 | 0.04499 |
| 302 | *Bacteroides* | -0.25 | 0.04567 |
| 303 | *Levilactobacillus* | -0.25 | 0.04846 |
| 304 | *Parabacteroides* | -0.25 | 0.04859 |
| 305 | *Proteiniphilum* | -0.25 | 0.04974 |

**Table S2** Correlation between bacteria and AA of different seasons.

| No | *Genus* | R | p |
| --- | --- | --- | --- |
| 1 | *Pantoea* | -0.45 | 0.00026 |
| 2 | *Lentimicrobium* | -0.42 | 0.00079 |
| 3 | *Companilactobacillus* | -0.41 | 0.00118 |
| 4 | *unclassified_Enterobacterales* | -0.31 | 0.01588 |
| 5 | *unclassified_Clostridiales_Incertae Sedis XI* | -0.31 | 0.01699 |
| 6 | *Sedimentibacter* | -0.31 | 0.01727 |
| 7 | *unclassified_Thermoactinomycetaceae 1* | -0.29 | 0.02410 |
| 8 | *Streptococcus* | -0.27 | 0.03572 |
| 9 | *Faecalicoccus* | 0.26 | 0.04841 |
| 10 | *Proteiniphilum* | 0.27 | 0.03694 |
| 11 | *unclassified_Clostridiales* | 0.28 | 0.03304 |
| 12 | *Raoultibacter* | 0.28 | 0.03100 |
| 13 | *unclassified_Bacillaceae 2* | 0.28 | 0.02885 |
| 14 | *unclassified_Peptococcaceae 2* | 0.29 | 0.02651 |
| 15 | *Membranicola* | 0.30 | 0.02043 |
| 16 | *unclassified_Lactobacillales* | 0.30 | 0.01891 |
| 17 | *Advenella* | 0.31 | 0.01697 |
| 18 | *Lactococcus* | 0.31 | 0.01648 |
| 19 | *unclassified_Ruminococcaceae* | 0.32 | 0.01309 |
| 20 | *Nocardiopsis* | 0.32 | 0.01279 |
| 21 | *Proteiniclasticum* | 0.33 | 0.01115 |
| 22 | *Pseudomonas* | 0.33 | 0.01035 |
| 23 | *unclassified_Mycobacteriales* | 0.33 | 0.00955 |
| 24 | *unclassified_Firmicutes* | 0.34 | 0.00858 |
| 25 | *Bacillus* | 0.34 | 0.00749 |
| 26 | *Acinetobacter* | 0.34 | 0.00748 |
| 27 | *Limosilactobacillus* | 0.35 | 0.00564 |
| 28 | *unclassified_Bacillales* | 0.36 | 0.00490 |
| 29 | *Pseudogracilibacillus* | 0.36 | 0.00438 |
| 30 | *Verticiella* | 0.37 | 0.00398 |
| 31 | *Bacteroides* | 0.38 | 0.00280 |
| 32 | *Aquihabitans* | 0.39 | 0.00191 |
| 33 | *Synergistes* | 0.40 | 0.00150 |
| 34 | *Atopostipes* | 0.40 | 0.00145 |
| 35 | *unclassified_Staphylococcaceae* | 0.43 | 0.00054 |
| 36 | *unclassified_Peptostreptococcaceae* | 0.46 | 0.00026 |
| 37 | *unclassified_Bacteroidaceae* | 0.47 | 0.00016 |
| 38 | *unclassified_Marinilabiliaceae* | 0.48 | 0.00011 |
| 39 | *unclassified_Moraxellaceae* | 0.48 | 0.00011 |
| 40 | *unclassified_Bacteria* | 0.48 | 0.00009 |
| 41 | *unclassified_Micrococcales* | 0.49 | 0.00008 |
| 42 | *unclassified_Alcaligenaceae* | 0.50 | 0.00005 |
| 43 | *Macellibacteroides* | 0.50 | 0.00004 |
| 44 | *unclassified_Acidaminococcaceae* | 0.51 | 0.00003 |
| 45 | *unclassified_Comamonadaceae* | 0.52 | 0.00002 |
| 46 | *unclassified_Streptococcaceae* | 0.52 | 0.00002 |
| 47 | *unclassified_Bacteroidetes* | 0.53 | 0.00002 |
| 48 | *Sphaerochaeta* | 0.53 | 0.00001 |
| 49 | *Fermentimonas* | 0.55 | 0.00000 |
| 50 | *unclassified_Flavobacteriaceae* | 0.56 | 0.00000 |
| 51 | *unclassified_Bacilli* | 0.56 | 0.00000 |
| 52 | *Sporobacter* | 0.57 | 0.00000 |
| 53 | *unclassified_Lachnospiraceae* | 0.61 | 0.00000 |
| 54 | *unclassified_Betaproteobacteria* | 0.63 | 0.00000 |
